# Do sex steroids exert sex-specific and/or opposite effects on gene expression in lacrimal and meibomian glands?

**Published:** 2009-08-10

**Authors:** David A. Sullivan, Roderick V. Jensen, Tomo Suzuki, Stephen M. Richards

**Affiliations:** ^^1^^Schepens Eye Research Institute and Department of Ophthalmology, Harvard Medical School, Boston, MA; ^^2^^Department of Biological Sciences, Virginia Tech, Blacksburg, VA

## Abstract

**Purpose:**

We hypothesize that sex steroids induce sex-specific and/or opposite effects in the lacrimal and meibomian glands and that these actions may influence the prevalence of dry eye syndrome. The objective of this study was to begin to test this hypothesis.

**Methods:**

Lacrimal and meibomian glands were obtained from ovariectomized mice that had been treated with testosterone or control vehicle for 14 days. Samples were processed for the isolation of RNA, and analyzed for differentially expressed mRNAs using CodeLink Bioarrays and quantitative real-time PCR (qPCR) techniques. Data were compared to those obtained following testosterone treatment of orchiectomized mice, as well as after the administration of 17β-estradiol and/or progesterone to ovariectomized mice.

**Results:**

Our findings demonstrate that testosterone regulates the expression of thousands of genes in the lacrimal and meibomian glands of ovariectomized mice. The magnitude and extent of these hormonal effects, which encompassed numerous biological, molecular, and cellular ontologies, was tissue-dependent. Particularly notable was the androgen stimulation of meibomian gland genes related to lipid metabolic pathways, and the suppression of genes associated with keratinization. Many of the genes regulated by testosterone in female tissues were identical to those controlled by androgens in male lacrimal and meibomian glands. However, some genes were modulated in a sex-specific manner. In addition, a number of the androgen-regulated genes in female glands were altered in the opposite direction by 17β-estradiol and/or progesterone.

**Conclusions:**

Our results support our hypothesis that sex steroids may induce sex-specific and/or opposite effects in the lacrimal and meibomian glands. Whether these actions contribute to the prevalence of dry eye remains to be determined.

## Introduction

Sex steroid hormones, such as androgens and estrogens, exert significant effects on almost every cell, tissue, and organ system of the body [1]. However, these hormone actions may not be the same in both males and females. Rather, sex steroid influence may be sex-specific. For example, sex steroids elicit sex-specific effects on many tissues (e.g. hippocampus, spinal cord, vasculature, muscle, and liver) and cells (e.g. preadipocytes, neutrophils, and antigen-presenting cells) [2-9]. Sex steroids may also induce opposite [10-14], and even antagonistic [15], influences, such as on sebaceous gland activity, cerebrovascular function, microtubule polymerization, chronic allograft nephropathy, autoimmunity, and the cystic fibrosis transmembrane conductance regulator (i.e. a chloride channel). These differential actions of sex steroids in males and females may contribute to a variety of conditions, including stress, atherosclerosis, coronary heart disease, fat distribution, inflammation, autoimmune disease, infection, pain syndromes, stroke, and lung development [2-5,8-10,14].

We hypothesize that sex-specific and/or opposite effects of sex steroids also occur in the lacrimal and meibomian glands and may influence the prevalence of dry eye disease. In support of this hypothesis, investigators have reported that sex steroid actions on the transcription of certain genes, the translation of the corresponding proteins, and the development of paradoxical inflammation in the lacrimal gland are sex-biased [15-21]. Moreover, we have discovered that sex and sex steroid hormones are critical factors in the pathogenesis of dry eye syndromes [22-31], which occur predominantly in women [32]. We have also discovered that androgens may suppress, and estrogens may promote, aqueous-deficient and/or evaporative dry eye [22-26,31]. We hypothesize that these opposing sex steroid actions may involve antagonist effects between androgens and estrogens in the lacrimal and meibomian glands.

However, whether sex steroids exert sex-specific or antagonistic effects in ocular adnexal tissues is unknown. The purpose of this study was to determine whether sex steroids do elicit sex-specific and/or opposite effects in both the lacrimal and meibomian glands. Towards this end, we examined the impact of testosterone on glandular gene expression in ovariectomized mice, and compared these effects to those found in androgen-treated orchiectomized mice. We also compared the testosterone-induced gene alterations in the female lacrimal and meibomian glands to those elicited by 17β-estradiol, progesterone, or both hormones together in these tissues.

## Methods

### Animals and hormone treatment

Age-matched and young adult BALB/c mice, that were ovariectomized at 8 weeks of age, were obtained from Taconic Laboratories (Germantown, NY). Animals were housed in constant temperature rooms with fixed light/dark intervals of 12 h duration. Ten days after surgery, pellets containing vehicle (cholesterol, methylcellulose, lactose) or testosterone (10 mg) were implanted subcutaneously in the ovariectomized mice. The pellets were purchased from Innovative Research of America (Sarasota, FL) and were designed for the constant release of placebo or physiological amounts of androgen (for a male [33-36]) for 3 weeks. After 14 days of treatment, mice (n=5-6 mice/ condition/ experiment) were sacrificed by CO__2__ inhalation and exorbital lacrimal and meibomian glands were removed. The meibomian glands were excised from the upper and lower lids under direct visualization with a biomicroscope. This surgical procedure involved making a small incision near the inner corner of the eyelid, separating skin and subcutaneous tissue from the inner to outer aspect of the lid, and then removing skin from the meibomian glands by cutting at the mucocutaneous junction. Following these steps, the palpebral conjunctiva was removed from the meibomian glands, and the glands were dissected from the remaining tissue by starting at the outer lid corner and carefully avoiding an adjacent vein. Tissues were pooled according to group (n=10-12 glands/ sample) and processed for RNA analysis. All studies with mice were approved by the Institutional Animal Care and Use Committee of The Schepens Eye Research Institute (Boston, MA) and adhered to the Association for Research in Vision and Ophthalmology Statement for the Use of Animals in Ophthalmic and Vision Research.

### Molecular biological procedures

To determine the influence of testosterone on lacrimal and meibomian gland gene expression, total RNA was extracted from tissues by using TRIzol reagent (Invitrogen Corp., Carlsbad, CA). Lacrimal tissue RNA was further purified with RNAqueous spin columns (Ambion, Austin, Tx). Glandular RNA samples were exposed to RNase-free DNase (Invitrogen), examined spectrophotometrically at 260 nm to determine concentration and analyzed with a RNA 6000 Nano LabChip and an Agilent 2100 Bioanalyzer (Agilent Technologies, Palo Alto, CA) to verify RNA integrity.

Gene expression was assessed by using CodeLink Uniset Mouse I Bioarrays (~10,000 genes; Amersham Biosciences/GE Healthcare, Piscataway, NJ). The RNA samples were processed for CodeLink Bioarray hybridization, according to published procedures [37]. In brief, cDNA was synthesized from RNA (2 µg) with a CodeLink Expression Assay Reagent Kit (Amersham, Piscataway, NJ) and purified with a QIAquick purification kit (Qiagen, Valencia, CA). Samples were then dried, and cRNA was produced with a CodeLink Expression Assay Reagent Kit (Amersham), recovered with an RNeasy kit (Qiagen) and quantified with an UV spectrophotometer. Fragmented, biotin-labeled cRNA was incubated and shaken (300 rpm shaker) on a CodeLink Bioarray at 37 ^°^C for 18 h. After this time period, the Bioarray was washed, exposed to streptavidin-Alexa 647, and scanned by utilizing ScanArray Express software and a ScanArray Express HT scanner (Packard BioScience, Meriden, CT) with the laser set at 635 nm, laser power at 100%, and photomultiplier tube voltage at 60%. Scanned image files were appraised by utilizing CodeLink image and data analysis software (Amersham), which yielded both raw and normalized hybridization signal intensities for each array spot. The spot intensities (~10,000 ) on the microarray image were standardized to a median of 1. Normalized data, with signal intensities exceeding 0.50 for the meibomian gland and 0.75 for the lacrimal gland, were evaluated with GeneSifter.net software (Geospiza, Seattle, WA). This comprehensive program also generated gene ontology and z-score reports. These ontologies encompassed biological processes, molecular functions and cellular components and were organized according to the guidelines of the Gene Ontology Consortium [38]. Gene expression data were analyzed without and with log transformation and statistical evaluation of these data was performed with Student’s t-test (two-tailed, unpaired) with GeneSifter.net software. Genes that were expressed in similar or opposite directions in different experiments were identified by using the GeneSifter.net intersector program (Geospiza). The data from the individual Bioarrays (n=6) are accessible for download through the National Center for Biotechnology Information’s Gene Expression Omnibus (GEO) via series accession number GSE3995.

### Real time PCR procedures

The differential expression of selected genes was confirmed by using quantitative real-time PCR (qPCR), as previously described [33,39]. In brief, sense and anti-sense primers were designed by utilizing Primer Express Software, version 1.5a (Applied Biosystems, Inc., Foster City, CA). The qPCR reactions were performed with Applied Biosystems’ SYBR Green PCR Master Mix, MicroAmp Optical 96-Well Reaction Plates, ABI PRISM Optical Adhesive Covers and the GeneAmp 7900 HT Sequence Detection System. Gene expression was calculated by using either the Relative Standard Curve Method or the Comparative C__T__ Method, and standardizing levels to that of glyceraldehyde-3-phosphate dehydrogenase or tubulin δ1 mRNA. Dissociation curves were examined to ensure the absence of secondary PCR products.

## Results

### Androgen influence on gene expression in female lacrimal and meibomian glands

To determine the influence of testosterone on lacrimal and meibomian gland gene expression, tissues were obtained from placebo- and androgen-treated, ovariectomized mice (n=5-6/group/ experiment) and processed for analysis by utilizing CodeLink Uniset Mouse I Bioarrays. Examination of non- and log-transformed data from 3 separate experiments demonstrated that testosterone significantly altered the expression of thousands of genes in female ocular tissues. Androgen action significantly upregulated 768 genes (e.g. transforming growth factor β1 and β3), and significantly downregulated 1,350 genes (e.g. pancreatic lipase related protein 1; Table 1 and Table 2), in the lacrimal gland. Moreover, testosterone administration increased the expression of 726 genes (e.g. glutathione peroxidase 3) and decreased the activity of 283 genes (e.g. small proline-rich protein 2A) in the meibomian gland (Table 1 and Table 3). The magnitude of these hormonal effects was tissue-dependent. Testosterone treatment induced a ≥10 fold change in the expression of over 45 lacrimal gland genes, and elicited a ≥2 fold response in the activity of 23 meibomian gland genes.

**Table 1:** Influence of testosterone on gene expression in the female lacrimal and meibomian glands.

	**Genes ↑**	**Genes ↓**	**Total**
**Lacrimal gland**			
No transformation	704	1,286	1,990
Log transformation	725	1,304	2,029
Total	768	1,350	2,118
**Meibomian gland**			
No transformation	697	259	956
Log transformation	693	273	966
Total	726	283	1,009

Data were evaluated with and without log transformation. The numbers of common and non-overlapping genes between analytical categories were determined, and then the total numbers were calculated. The expression of listed genes was significantly (p <0.05) up (↑)- or down (↓)-regulated by testosterone treatment.

**Table 2:** Testosterone effect on gene expression ratios in the female lacrimal gland.

**Accession number**	**Gene**	**Ratio**	**p value**	**Ontology**
**Testosterone>Placebo**				
NM_019515	neuromedin	975.7	0.0003	cell communication
AF071068	dopa decarboxylase	86.2	0.0005	amino acid metabolism
NM_008957	patched homolog 1	64.5	0.0000	regulation of growth
NM_009021	retinoic acid induced 1	40.6	0.0033	transcription
NM_010643	kallikrein 1-related peptidase b24	38.8	0.0001	proteolysis
NM_053178	acyl-CoA synthetase bubblegum family member 1	37.6	0.0023	very-long-chain fatty acid metabolic process
NM_007936	Eph receptor A4	29.4	0.0001	neurogenesis
NM_007413	adenosine A2b receptor	28.2	0.0001	signal transduction
NM_007556	bone morphogenetic protein 6	27.2	0.0082	transmembrane receptor protein serine/threonine kinase signaling pathway
AK007577	chordin-like 2	27.2	0.0005	transmembrane receptor protein serine/threonine kinase signaling pathway
**Placebo>Testosterone**				
NM_018874	pancreatic lipase related protein 1	81.1	0.0001	metabolism
NM_018781	early growth response 3	19.8	0.0401	transcription
NM_009714	asialoglycoprotein receptor 1	15.7	0.0118	vesicle-mediated transport
NM_023186	chitinase, acidic	15.1	0.0480	polysaccharide catabolic process
NM_008456	kallikrein 1-related peptidase b5	15.1	0.0288	proteolysis
NM_007446	amylase 1, salivary	12.5	0.0418	carbohydrate metabolic process
NM_011105	polycystin	14.8	0.0015	cell communication
AK002477	plasma membrane proteolipid	12.0	0.0004	transport
NM_022305	UDP-Gal:betaGlcNAc beta 1,4- galactosyltransferase, polypeptide 1	9.2	0.0003	metabolism
NM_007409	alcohol dehydrogenase 1 (class I)	9.1	0.0049	metabolism

The accession number is the sequence identity of the gene fragment expressed on the CodeLink Bioarray. This sequence appears in the nucleotide database of the National Center for Biotechnology Information (NCBI). Relative ratios were calculated by comparing the degree of gene expression in lacrimal glands from placebo- and testosterone-treated ovariectomized mice. Genes listed had a signal intensity average of >5.0 in at least one group, a comparative p value (between glands) of <0.05 and a known identity. These ratios were generated from non- and log-transformed data.

**Table 3:** Testosterone influence on gene expression ratios in the female meibomian gland.

**Accession number**	**Gene**	**Ratio**	**p value**	**Ontology**
**Testosterone>Placebo**				
NM_030265	Kv channel interacting protein 4	8.8	0.0134	transport
NM_011351	sema domain, transmembrane domain, and cytoplasmic domain, (semaphorin) 6C	4.0	0.0036	multicellular organismal development
NM_008161	glutathione peroxidase 3	3.0	0.0063	response to oxidative stress
NM_008362	interleukin 1 receptor, type I	2.4	0.0021	signal transduction
NM_026523	neuromedin B	2.3	0.0019	neuropeptide signaling pathway
NM_008288	hydroxysteroid 11β dehydrogenase 1	2.2	0.0123	lipid metabolic process
NM_008324	indoleamine-pyrrole 2,3 dioxygenase	2.2	0.0380	positive regulation of T cell tolerance induction
AA832579	a disintegrin-like and metallopeptidase with thrombospondin type 1 motif, 2	2.2	0.0003	proteolysis
NM_010174	fatty acid binding protein 3	2.2	0.0409	phosphatidylcholine biosynthetic process
U08020	collagen, type I, α1	2.2	0.0004	negative regulation of cell-substrate adhesion
**Placebo>Testosterone**				
NM_007894	eosinophil-associated, ribonuclease A family, member 1	4.3	0.0016	nucleic acid binding
NM_011468	small proline-rich protein 2A	3.3	0.0082	regulation of cell shape
NM_013650	S100 calcium binding protein A8	2.8	0.0332	chemotaxis
NM_022984	resistin	2.4	0.0136	hormone activity
NM_009154	sema domain, seven thrombospondin repeats, trans-membrane domain and short cytoplasmic domain	2.1	0.0077	multicellular organismal development
AK005197	kin of IRRE like 3	2.0	0.0003	hemopoiesis
NM_010449	homeo box A1	1.9	0.0441	transcription
NM_008620	guanylate binding protein 4	1.9	0.0231	biological_process
NM_031184	GLIS family zinc finger 2	1.9	0.0206	transcription
AK011647	protein kinase, cAMP dependent regulatory, type IIβ	1.9	0.0021	regulation of protein amino acid phosphorylation

Relative ratios were determined by comparing the degree of gene expression in meibomian glands from placebo- and testosterone-treated ovariectomized mice. Genes listed had a comparative p value (between glands) of <0.05, as well as a known identity. These ratios were generated from non- and log-transformed data.

The influence of androgen exposure was particularly notable in its significant stimulation of such ontologies as cell cycle, transferase activity and chromosomes in the lacrimal gland (Table 4), and protein transport, oxidoreductase activity and mitochondria in the meibomian gland (Table 5). Testosterone treatment was also associated with a significant suppression of pathways linked to translation, structural molecule activity and ribosomes in lacrimal tissue, and the immune response, receptor binding and plasma membranes in the meibomian gland. Of particular interest was the significant androgen impact on the expression of genes linked to multiple lipid metabolic processes (e.g. biosynthesis, transport, oxidation) in meibomian tissue (Table 6). Also striking was the testosterone suppression of genes related to keratinization (z score=10.2) and cell fate commitment (z score=3.2) in the meibomian gland (Table 7).

**Table 4:** Impact of testosterone on the expression of gene ontologies in the female lacrimal gland.

**Ontology**	**Testosterone genes ↑**	**Placebo genes ↑**	**Testosterone z-score**	**Placebo z-score**
**Biological process**				
cell cycle	54	48	**4.42**	-1.43
mitotic cell cycle	25	18	**4.02**	-0.94
DNA metabolic process	35	25	**3.78**	-2.01
protein metabolic process	98	248	**-2.06**	2.45
anatomical structure development	81	173	**-2.51**	-1.66
multicellular organismal process	119	231	**-2.52**	-2.88

translation	5	62	-2.64	**7.28**
cellular protein metabolic process	96	242	-2.02	**2.36**
proteolysis	30	89	-1.59	**2.06**
protein amino acid phosphorylation	30	36	-0.44	**-3.47**
signal transduction	113	152	0.67	**-4.06**
cell communication	122	169	0.14	**-4.59**
**Molecular function**				
transferase activity, transferring acyl groups	15	5	**3.18**	-2.35
small GTPase regulator activity	13	8	**2.47**	-1.38
vitamin binding	10	12	**2.07**	0.73
endopeptidase activity	8	38	**-2.04**	1.88
calcium ion binding	24	71	**-2.2**	0.59

structural constituent of ribosome	5	51	-0.8	**11.45**
structural molecule activity	12	75	-1.62	**7.63**
hydrogen ion transmembrane transporter activity	5	18	0.73	**4.68**
ribonucleotide binding	65	95	-0.53	**-3.21**
signal transducer activity	64	91	-0.37	**-3.24**
transferase activity	83	97	1.63	**-3.28**
**Cellular component**				
chromosome	28	11	**3.91**	-3.17
nucleolus	13	18	**2.84**	2.1
nucleus	207	329	**2.43**	-0.23
plasma membrane part	40	77	**-2.25**	-2.76

ribosome	6	59	-1.15	**10.91**
ribonucleoprotein complex	13	85	-1.69	**8.59**
macromolecular complex	80	228	-1.12	**5.79**
chromosomal part	22	9	3.39	**-2.77**
intrinsic to membrane	163	281	-0.89	**-2.58**
membrane	230	417	-1.46	**-2.43**

Specific ontologies, with some of the highest and lowest z-scores, were selected after the analysis of non-transformed data. A z-score is a statistical rating of the relative expression of gene ontologies, and shows how much each ontology is over- or under-represented in a given gene list. In other words, the z-score is a standardized difference using the expected value and standard deviation of the number of genes meeting the criterion of a gene ontology term under a hypergeometric distribution [97]. Positive z scores represent gene ontology terms with a greater number of genes meeting the criterion than is expected by chance, whereas negative z scores reflect gene ontology terms with fewer genes meeting the criterion than expected by chance. A z score close to zero indicates that the number of genes meeting the criterion approximates the expected number [97]. Z-scores with values >2.0 or less than <-2.0, and with ≥20 genes, are shown for selected ontologies. High and low values for the placebo and testosterone groups in designated ontologies are highlighted in bold print. Terms: Testosterone genes ↑ - number of genes up-regulated in lacrimal glands of testosterone-treated mice, as compared to those of the ‘placebo’ group; Placebo genes ↑ - number of genes up-regulated in lacrimal glands of placebo-treated mice, relative to those of the ‘testosterone’ group; z-score - specific score for the up-regulated genes in the placebo- and hormone-treated lacrimal glands.

**Table 5:** Influence of testosterone on the expression of gene ontologies in the female meibomian gland.

**Ontology**	**Testosterone genes ↑**	**Placebo genes ↑**	**Testosterone z-score**	**Placebo z-score**
**Biological Process**				
cellular catabolic process	66	12	**5.53**	-0.5
oxidation reduction	53	5	**5.46**	-1.72
protein transport	45	3	**3.44**	-2.49
cell communication	72	48	**-4.85**	0.47
signal transduction	58	41	**-5.16**	0.16
regulation of biological process	151	96	**-6.67**	1.34

immune response	16	24	-1.26	**5.6**
organ development	40	50	-4.34	**4.43**
response to stimulus	56	51	-3.32	**3.68**
protein transport	45	3	3.44	**-2.49**
protein localization	49	4	3.15	**-2.53**
macromolecule localization	51	4	3.25	**-2.62**
**Molecular Function**				
oxidoreductase activity	57	8	**5.81**	-0.88
catalytic activity	258	58	**5.36**	-2.7
peptidase activity	33	10	**2.4**	0.51
DNA binding	39	35	**-4.57**	1.33
molecular transducer activity	31	32	**-4.63**	1.56
signal transducer activity	31	32	**-4.63**	1.56

receptor binding	18	19	-2.05	**2.59**
receptor activity	23	29	-4.5	**2.05**
nucleotide binding	80	19	0.06	**-2.28**
**Cellular Component**				
mitochondrion	82	11	**6.19**	-1.2
cytoplasm	341	76	**6.1**	-2.67
organelle membrane	59	10	**5.64**	-0.18
cytoskeleton	21	15	**-2.76**	0.62
nucleus	149	65	**-3.02**	0.15
plasma membrane	53	51	**-6.25**	2.21

extracellular space	8	16	-2.57	**3.83**
plasma membrane part	20	33	-5.21	**3.22**
extracellular region part	29	19	-0.44	**2.49**
intracellular part	425	124	2.81	**-2.17**
cytoplasmic part	235	40	7.44	**-2.47**
cytoplasm	341	76	6.1	**-2.67**

Specific ontologies, with some of the highest and lowest z-scores, were selected following the analysis of non-transformed data. Criteria for inclusion in the Table were an ontology containing ≥20 genes and having a z-score >2.0 or <-2.0. High and low values for the placebo and testosterone groups in designated ontologies are highlighted in bold print.

**Table 6:** Androgen effect on genes associated with lipid metabolic processes in the female meibomian gland.

**Up-regulation**	**Down-regulation**
3-hydroxy-3-methylglutaryl-Coenzyme A reductase	Arachidonate 15-lipoxygenase
Acyl-CoA synthetase medium-chain family member 3	ATPase, Na+/K+ transporting, α1 polypeptide
Acyl-CoA thioesterase 12	Enoyl-Coenzyme A, hydratase/3-hydroxyacyl
Acyl-Coenzyme A oxidase 2, branched chain	Coenzyme A dehydrogenase
Adipose differentiation related protein	Farnesyl diphosphate farnesyl transferase 1
Aldehyde dehydrogenase family 1, subfamily A1	Fatty acid binding protein 5, epidermal
Apolipoprotein C-II	HNF1 homeobox A
Apolipoprotein E	Interleukin 1β
Arachidonate 12-lipoxygenase	Phospholipase A2, group IIF
Dodecenoyl-Coenzyme A δ isomerase (3,2 trans-enoyl-Coenyme A isomerase)	Phospholipase D1
Dolichol-phosphate (βD) mannosyltransferase 1	Protein kinase, cAMP dependent regulatory, type IIβ
Elongation of very long chain fatty acids (FEN1/Elo2, SUR4/Elo3, yeast)-like 3	Sortilin-related receptor, LDLR class A repeats-containing
Fatty acid binding protein 3, muscle and heart	Steroid sulfatase
Glycerol-3-phosphate acyltransferase, mitochondrial	
Hexosaminidase A	
Hexosaminidase B	
Hydroxysteroid (17β) dehydrogenase 12	
Hydroxysteroid (17 β) dehydrogenase 2	
Hydroxysteroid (17 β) dehydrogenase 7	
Hydroxysteroid 11 β dehydrogenase 1	
LAG1 homolog, ceramide synthase 4	
Mitochondrial trans-2-enoyl-CoA reductase	
N-acylsphingosine amidohydrolase 2	
Oxysterol binding protein-like 5	
Peroxisome biogenesis factor 7	
Phenylalkylamine Ca2+ antagonist (emopamil) binding protein	
Phosphatidylserine synthase 1	
Prenyl (solanesyl) diphosphate synthase, subunit 1	
Prostaglandin E synthase 2	
RIKEN cDNA 0610007P14 gene	
Solute carrier family 27 (fatty acid transporter), member 4	
Sphingomyelin phosphodiesterase 1, acid lysosomal	
Sphingomyelin phosphodiesterase, acid-like 3A	
Sphingosine-1-phosphate phosphatase 1	
Stearoyl-Coenzyme A desaturase 1	
Stearoyl-coenzyme A desaturase 3	
Sulfotransferase family 1A, phenol-preferring, member 1	
Transmembrane 7 superfamily member 2	

Genes were significantly (p <0.05) up- or down-regulated by testosterone treatment.

**Table 7:** Androgen influence on genes linked to keratinization and cell fate commitment in the female meibomian gland.

**Up-regulation**	**Down-regulation**
**Keratinization**	**Keratinization**
	Small proline-rich protein 2A
	Small proline-rich protein 2B
	Small proline-rich protein 3
	Keratin 17
	Cornifelin
	Periplakin
	Transglutaminase 1, K polypeptide

**Cell fate commitment**	**Cell fate commitment**
POU domain, class 2, transcription factor 1	Notch gene homolog 2
Cell division cycle 42 homolog (S. cerevisiae)	Notch gene homolog 1
	Multiple endocrine neoplasia 1
	Delta-like 3
	GS homeobox 1
	Kinase insert domain protein receptor
	Sine oculis-related homeobox 1 homolog
	T-cell acute lymphocytic leukemia 1

Genes were significantly (p <0.05) up- or down-regulated by testosterone treatment.

To verify in part the CodeLink Bioarray results, selected genes were analyzed by qPCR. This experimental approach confirmed the effect of testosterone on all tested genes (Table 8).

**Table 8:** Confirmation of selected CodeLink Bioarray results.

**Accession number**	**Gene**	**CodeLink Ratios**	**qPCR Ratios**
**Lacrimal gland**			
**Testosterone>Placebo**			
NM_009735	β-2 Microglobulin	2.6; 3.4; 3.6	6.8; 9.4; 10.0
NM_011978	Solute carrier family 27, member 2	4.4; 3.7; 4.0	8.0; 10.0; 18.9
NM_009031	Retinoblastoma binding protein 7	4.0; 3.0; 3.2	4.4; 3.4; 4.8

**Placebo>Testosterone**			
NM_018874	Pancreatic lipase related protein 1	77.3; 91.3; 73.0	2,121; 680; 320
NM_009714	Asialoglycoprotein receptor 1	10.0; 21.3; 14.8	7.1; 35.9; 52.1
NM_010726	Phytanoyl-CoA-hydroxylase	6.1; 7.1; 5.8	13.1; 13.2; 20.4

**Meibomian gland**			
**Testosterone>Placebo**			
NM_008161	Glutathione peroxidase 3	3.6; 3.0; 2.2	5.7; 3.8; 5.0
NM_026523	Neuromedin B	2.5; 2.3; 1.9	2.1; 1.8; 1.5

**Placebo>Testosterone**			
NM_011468	Small proline-rich protein 2A	3.9; 3.6; 2.5	5.3; 7.3; 4.9
NM_022984	Resistin	2.0; 2.1; 2.9	3.6; 2.9; 2.5

The expression of designated genes, that were shown to be significantly altered in lacrimal and meibomian glands of placebo- or testosterone-treated mice by using CodeLink Biooarrays, were re-examined with qPCR procedures. The relative ratios of gene expression in 3 separate experiments are listed in the CodeLink and qPCR “Ratios” columns.

Surprisingly, the effects of testosterone on the lacrimal and meibomian gland were not entirely tissue-specific. As shown in Table 9, androgen treatment caused analogous changes in the expression of 127 genes in both tissues, and opposite responses in 165 genes. Genes regulated in the same manner included vascular endothelial growth factor A, hyaluronan mediated motility receptor, and lipocalin 3 (Table 10). Those controlled in a different way included chemokine binding protein 2, cholecystokinin, and matrix metalloproteinase 3 (Table 10).

**Table 9:** Similar and opposite effects of testosterone on gene expression in female lacrimal and meibomian glands.

**Testosterone effect**	**Genes - no transform**	**Genes - log transform**	**Total genes**
Lacrimal Gland ↑, Meibomian Gland ↑	58	54	59
Lacrimal Gland ↑, Meibomian Gland ↓	26	30	31
Lacrimal Gland ↓, Meibomian Gland ↑	153	141	154
Lacrimal Gland ↓, Meibomian Gland ↓	64	67	68

Data were analyzed with and without log transformation (“Transform”). The expression of the same genes was significantly (p <0.05) up (↑)- or down (↓)-regulated by androgen treatment of ovariectomized mice.

**Table 10:** Testosterone effect on specific genes in the lacrimal and meibomian glands.

**Accession number**	**Gene**	**LG % ↑**	**LG % ↓**	**MG % ↑**	**MG % ↓**
NM_009127	stearoyl-Coenzyme A desaturase 1	53		24	
NM_009505	vascular endothelial growth factor A	50		8	
NM_010555	interleukin 1 receptor, type II	82		36	
NM_011026	purinergic receptor P2X, ligand-gated ion channel 4	38		34	
NM_013552	hyaluronan mediated motility receptor	640		22	
NM_013754	insulin-like 6	433		49	
NM_007470	apolipoprotein D		88		29
NM_010483	5-hydroxytryptamine (serotonin) receptor 5B		30		29
NM_010694	lipocalin 3		199		25
NM_013706	CD52 antigen		88		19
NM_013822	jagged 1		181		27
NM_018766	neurotensin receptor		38		34
AK010367	dynamin binding protein	528			67
NM_021489	coagulation factor XII (Hageman factor)	233			77
NM_021609	chemokine binding protein 2	240			26
NM_023580	Eph receptor A1	55			12
NM_023907	forkhead box I1	18			15
NM_008351	interleukin 12a	110			45
NM_009801	carbonic anhydrase 2		30	13	
NM_031161	cholecystokinin		161	23	
NM_017370	haptoglobin		218	105	
NM_010809	matrix metalloproteinase 3		197	81	
NM_007740	procollagen, type IX, alpha 1		35	16	
NM_009425	tumor necrosis factor (ligand) superfamily, member 10		104	30	

Genes were significantly (p <0.05) increased (↑) or decreased (↓) by testosterone treatment in the lacrimal (LG) and meibomian (MG) glands of ovariectomized mice. Numbers equal the percentage (%) increase or decrease, relative to the placebo-treated control level.

### Comparative effects of androgens on gene expression in male and female lacrimal and meibomian glands

We have previously discovered that testosterone treatment significantly influences the expression of over 2,000 and 1,500 genes in the lacrimal and meibomian glands, respectively, of orchiectomized mice [40]. To examine whether these androgen actions are analogous to those found in ovariectomized mice, we compared the data from the earlier studies (n=10-14 glands/ sample/ experiment; n=3 experiments/ study) with orchiectomized mice to those in the present investigation. All gene expression results were generated with CodeLink Uniset Mouse I Bioarrays and analyzed with GeneSifter software.

Our analysis of log transformed data demonstrated that testosterone administration significantly altered the expression of many of the same genes in both ovariectomized and orchiectomized mice. This hormonal control included 1,072 similar genes in lacrimal glands and 285 similar genes in meibomian glands. The nature (i.e. up- or down-regulation) of the androgen effects was identical in 95.5% of the lacrimal gland genes, and 77.6% of the meibomian gland genes.

To further compare testosterone influence on glandular gene expression in female and male mice, we focused on genes in female tissues with relatively high array intensities, expression ratios (i.e. compared to the placebo-treated group), and significant differences (i.e. versus placebo). These more stringent criteria (lacrimal=p <0.001; intensity >2.0; ratio >1.8; meibomian=p <0.01; intensity >1.0; ratio >1.35) identified 173 (8.7% of the total) and 80 (8.4% of the total) genes in female lacrimal and meibomian glands, respectively. Comparative analyses showed that, with the exception of 4 genes, all lacrimal gland genes were increased or decreased in the same direction in males. The 4 exceptions were genes that were significantly reduced (ratios between 2.5 to 3.6 fold) by testosterone in female mice, but were not lower following androgen exposure in male lacrimal tissues (Table 11). In the meibomian gland the expression of 2 genes, that were significantly decreased (ratios=1.4 fold) in female tissues, were significantly increased (ratios=1.3 fold) in males (Table 11). Another 13 genes in the female meibomian gland, that showed significant expression differences (ratios between 1.4 to 1.8 fold) after testosterone exposure, were not influenced in the same direction in males (Table 11). Overall, these comparisons did not reveal any gene that was unique to female lacrimal or meibomian glands.

**Table 11:** Comparative effects of testosterone on gene expression in female and male glands.

**Genes significantly increased by testosterone in female, but not increased in male**	**Genes significantly decreased by testosterone in female, but not decreased in male**	**Genes significantly decreased by testosterone in female, but significantly increased in male**
**Lacrimal gland**	**Lacrimal gland**	**Lacrimal gland**
	Adult male medulla oblongata clone:6330419D11	
	homer homolog 3	
	potassium voltage-gated channel, shaker-related subfamily, member 2	
	lymphocyte antigen 78	

**Meibomian gland**	**Meibomian gland**	**Meibomian gland**
prostaglandin E synthase 2	glial fibrillary acidic protein	keratin associated protein 3-1
estrogen related receptor a	bone morphogenetic protein 8b	hypothetical protein, clone 1-53
neural proliferation, differ-entiation and control gene 1	interleukin 1β	
procollagen, type VI, α1	epithelial membrane protein 3	
tropomodulin 4	fatty acid binding protein 5, epidermal	
	chemokine (C-C motif) ligand 7	
	interleukin 1 family, member 6	

All genes were significantly (p <0.05) up- or down-regulated by testosterone treatment in ovariectomized mice. The female genes in not transformed data had the following characteristics: lacrimal gland=p <0.001, intensity >2.0, ratio >1.8; meibomian gland=p <0.01, intensity >1.0, ratio >1.35.

Additional comparisons demonstrated that many of the androgen effects on biological process, molecular function and cellular component ontologies in lacrimal and meibomian glands were the same in both females and males (data not shown).

### Comparative effects of testosterone, 17β-estradiol and/or progesterone on gene expression in female lacrimal and meibomian glands

We have found that 17β-estradiol and/or progesterone administration for 2 weeks significantly alters the expression of hundreds of genes in the lacrimal and meibomian glands of ovariectomized mice [41,42] (Table 12). To determine whether these sex steroid actions are unique, or the same as, or opposite to, those elicited by testosterone exposure, we compared the data from our previous investigations (n=14 glands/ sample/ experiment; n=3 experiments/ study) [41,42] to those in the current study. The ages of the BALB/c mice used in all experiments were similar. In addition, all gene expression results were obtained with CodeLink Uniset Mouse I Bioarrays and analyzed with GeneSifter software.

**Table 12:** Similarities and differences between the numbers of genes regulated by testosterone, 17β-estradiol and progesterone in the lacrimal and meibomian glands of ovariectomized mice.

**Sex steroid effect on gene expression**	**Lacrimal gland**	**Meibomian gland**
Testosterone ↑	725	693
Testosterone ↓	1304	273

17β-Estradiol ↑	175	82
17β -Estradiol ↓	188	87

Progesterone ↑	93	25
Progesterone ↓	137	134

17β -Estradiol + Progesterone ↑	144	101
17β -Estradiol + Progesterone ↓	198	188

17β -Estradiol ↑, Testosterone ↑	13	12
17β -Estradiol ↓, Testosterone ↓	17	5
17β -Estradiol ↑, Testosterone ↓	24	1
17β -Estradiol ↓, Testosterone ↑	42	13
		
Progesterone ↑, Testosterone ↑	9	0
Progesterone ↓, Testosterone ↓	13	11
Progesterone ↑, Testosterone ↓	2	0
Progesterone ↓, Testosterone ↑	19	12

17β -Estradiol + Progesterone ↑, Testosterone ↑	17	12
17β -Estradiol + Progesterone ↓, Testosterone ↓	29	11
17β -Estradiol + Progesterone ↑, Testosterone ↓	30	4
17β -Estradiol + Progesterone ↓, Testosterone ↑	46	23

The number of genes significantly (p <0.05) up (↑)- or down (↓)-regulated by steroid treatment of castrated mice is shown in the columns entitled “Lacrimal gland” and “Meibomian gland.” Results were obtained by comparing log-transformed data. The 17β-estradiol and progesterone data originated from other studies [42,43], for which the Association for Research in Vision and Ophthalmology is the copyright holder.

As shown in Table 12 and Table 13, the majority of genes regulated by testosterone, 17β-estradiol and/or progesterone in the lacrimal and meibomian glands were unique (i.e. hormone-specific). Treatment with 17β-estradiol, progesterone, or both sex steroids in combination significantly influenced less than 6.4% of genes controlled by androgens (Table 13). Conversely, testosterone significantly influenced less than 23.3% of estrogen- and progestin-regulated genes (Table 13).

**Table 13:** Percentages of the same genes influenced by testosterone, 17β-estradiol and/or progesterone in the lacrimal and meibomian glands of ovariectomized mice

**Sex steroid effect on** **gene expression**	**Lacrimal gland**		**Meibomian gland**	
	**E2±Prog/ Test %**	**Test/E2± Prog %**	**E2±Prog/ Test %**	**Test/E2± Prog %**
E2↑, Test↑	1.8	7.4	1.7	14.6
E2↓, Test↓	1.3	9.0	1.8	5.7
E2↑, Test↓	1.8	13.7	0.004	1.2
E2↓, Test↑	5.8	22.3	1.9	14.9

Prog↑, Test↑	1.2	9.7	0	0
Prog↓, Test↓	1.0	9.5	4.0	8.2
Prog↑, Test↓	0.002	2.2	0	0
Prog↓, Test↑	2.6	13.9	1.7	9.0

E2+ Prog↑, Test↑	2.3	11.8	1.7	16.8
E2+ Prog↓, Test↓	2.2	14.6	4.0	5.9
E2+ Prog↑, Test↓	2.3	20.8	1.5	4.0
E2+ Prog↓, Test↑	6.3	23.2	3.3	12.2

The percentages were calculated by dividing the number of genes significantly (p <0.05) up (↑)- or down (↓)-regulated by a given sex steroid treatment by the number of genes significantly (p <0.05) influenced by a different hormone(s), and multiplying that fraction by 100. The gene numbers used for these calculations are reported in Table 12. Abbreviations: E2=17β-estradiol; Prog=progesterone; Test=testosterone

However, although the total number of common genes was limited, the nature of the hormone response to testosterone, as compared to 17β-estradiol, progesterone or both hormones together, was typically different. Between 62.3 to 68.8% of the sex steroid effects (i.e. testosterone versus other hormones) in the lacrimal gland were in the opposite direction. Moreover, between 45.2 to 54.0% of the hormone responses in the meibomian gland were in the opposite direction. Examples of estrogen- and progestin-regulated genes that were modulated in a similar or opposite manner relative to testosterone are shown in Table 14 and Table 15.

**Table 14:** Similar and opposite effects of testosterone, 17β-estradiol and progesterone on gene expression in the lacrimal glands of ovariectomized mice

**E2↑, Test↑**	**Prog↑, Test↑**	**E2 + Prog↑, Test↑**
Fas apoptotic inhibitory molecule 2	transmembrane channel-like gene family 4	monoacylglycerol O-acyltransferase 1
acyl-Coenzyme A dehydrogenase, short chain	adenosine A2b receptor	adenosine A2b receptor
CD151 antigen	cytokine receptor-like factor 1	cyclin B2
topoisomerase (DNA) IIα	cell division cycle 20 homolog	vascular endothelial growth factor A
1-acylglycerol-3-phosphate O-acyltransferase 3	splicing factor 3a, subunit 1	cyclin A2

**E2↓, Test↓**	**Prog↓, Test↓**	**E2 + Prog↓, Test↓**
calpain 9	calpain 9	calpain 9
cholinergic receptor, nicotinic, α poly-peptide 1	nidogen 1	preproenkephalin 1
preproenkephalin 1	biotinidase	lymphocyte antigen 78
caspase 6	solute carrier family 25 (mitochondrial carrier, adenine nucleotide translocator), member 4	Notch gene homolog 1
hephaestin	zinc finger protein 95	hephaestin

**E2↑, Test↓**	**Prog↑, Test↓**	**E2 + Prog↑, Test↓**
asialoglycoprotein receptor 1	glycerol phosphate dehydrogenase 2, mitochondrial	asialoglycoprotein receptor 1
CD14 antigen	ribosomal protein L6	leucine aminopeptidase 3
pancreatic lipase related protein 1		pancreatic lipase related protein 1
CD 81 antigen		aminoacylase 1
polycystin		polycystin

**E2↓, Test↑**	**Prog↓, Test↑**	**E2 + Prog↓, Test↑**
cyclin E1	proteasome 26S subunit, ATPase 3, interacting protein	dynamin binding protein
dynamin binding protein	sialyltransferase 7	interleukin 12a
interleukin 12a	CD3 antigen, zeta polypeptide	retinol dehydrogenase 6
neuregulin 4	UDP-Gal:betaGlcNAc beta 1,4-galactosyltransferase, polypeptide 6	solute carrier family 27 (fatty acid transporter), member 2
forkhead box P3	forkhead box P3	forkhead box P3

Genes were significantly (p <0.05) up (↑)- or down (↓)-regulated by a given sex steroid treatment. Abbreviations: E2=17β-estradiol; Prog=progesterone; Test=testosterone

**Table 15:** Analogous and opposite effects of testosterone, 17β-estradiol and progesterone on gene expression in the meibomian glands of ovariectomized mice

**E2↑, Test↑**	**Prog↑, Test↑**	**E2 + Prog↑, Test↑**
hydroxysteroid (17β) dehydrogenase 7		tocopherol (α) transfer protein
glutathione peroxidase 3		glutathione peroxidase 3
haptoglobin		FK506 binding protein 5
interleukin 1 receptor, type II		interleukin 1 receptor, type II
cholecystokinin		cholecystokinin

**E2↓, Test↓**	**Prog↓, Test↓**	**E2 + Prog↓, Test↓**
myotubularin related protein 4	homer homolog 3 (Drosophila)	G protein-coupled receptor, family C, group 5, member C
heat shock protein 2	uridine mono-phosphate synthetase	heat shock protein 2
chemokine (C-C motif) ligand 27	frizzled-related protein	interleukin 12a
RE1-silencing transcription factor (REST) co-repressor 1	fatty acid binding protein 5, epidermal	CD52 antigen
phospholipase D1	phospholipase D1	phospholipase D1

**E2↑, Test↓**	**Prog↑, Test↓**	**E2 + Prog↑, Test↓**
cytochrome P450, family 2, subfamily g, polypeptide 1		cytochrome P450, family 2, subfamily g, polypeptide 1
		double cortin and calcium/calmodulin-dependent protein kinase-like 1
		RIKEN cDNA 1810016I24 gene
		RIKEN cDNA 2700085M18 gene

**E2↓, Test↑**	**Prog↓, Test↑**	**E2 + Prog↓, Test↑**
preproenkephalin 1	cytochrome c oxidase, subunit VIb	secreted acidic cysteine rich glycoprotein
cathepsin K	amine oxidase, copper containing 3	vascular endothelial growth factor A
secreted acidic cysteine rich glycoprotein	gastric intrinsic factor	preproenkephalin 1
vascular endothelial growth factor A	proteasome 26S subunit, non-ATPase, 5	proteasome 26S subunit, non-ATPase, 5
matrix metallo-proteinase 3	mannoside acetyl-glucosaminyl-transferase 3	procollagen, type I, α1

Genes were significantly (p <0.05) up (↑)- or down (↓)-regulated by a given sex steroid exposure. Abbreviations: E2=17β-estradiol; Prog=progesterone; Test=testosterone.

## Discussion

Our findings demonstrate that testosterone regulates the expression of thousands of genes in the lacrimal and meibomian glands of ovariectomized mice. The magnitude and extent of these hormonal effects, which encompassed numerous biological, molecular and cellular ontologies, was tissue-dependent. Particularly notable was the androgen stimulation of meibomian gland genes related to lipid metabolic pathways, and the suppression of genes associated with keratinization. Many of the genes regulated by testosterone in female tissues were identical to those controlled by androgens in male lacrimal and meibomian glands. However, some genes were modulated in a sex-specific manner. In addition, a number of the androgen-regulated genes in female glands were altered in the opposite direction by 17β-estradiol and/or progesterone. Overall, our results support our hypothesis that sex steroids may induce sex-specific and opposite effects in the lacrimal and meibomian glands.

Our observation that testosterone influences the expression of multiple genes in female ocular adnexal tissues was anticipated. Androgens are known to exert a tremendous impact on the structure and function of the lacrimal gland, including such aspects as the cellular morphology, nuclear architecture, protein synthesis, enzyme activity, receptor expression and fluid and protein secretion [28]. Similarly, the meibomian gland is an androgen target organ, and androgens appear to regulate this tissue’s function (e.g. lipid production) [22-25,43-45]. Androgen deficiency, in turn, has been linked to lacrimal and meibomian gland dysfunction, and a corresponding aqueous tear deficiency and evaporative dry eye [22-26,28-30]. A number of these ocular effects, due to the presence or absence of androgens, may be associated with glandular alterations in gene activity [39,40].

Of the many androgen actions on gene expression in the lacrimal and meibomian glands, two that stand out are the stimulation of meibomian gland genes associated with lipid metabolic pathways, and the suppression of genes related to keratinization. First, the upregulation of genes involved with lipid biosynthesis, transport, and metabolism is reminiscent of testosterone’s similar influence on the male meibomian gland [39,46,47]. Androgens stimulate numerous lipid pathway genes in this tissue, including those related to lipogenesis, steroidogenesis, and cholesterogenesis [39,46,47]. Second, the testosterone downregulation of meibomian gland genes associated with keratinization may explain on a molecular level how androgens inhibit this process. Keratinization is believed to be a primary cause of meibomian gland dysfunction [48,49], which leads to tear film hyperosmolarity and instability and evaporative dry eye [50-53]. Androgens appear to prevent this keratinization, because androgen insufficiency (e.g. during anti-androgen treatment, complete androgen insensitivity syndrome and/or aging) is associated with keratinization of the meibomian gland ductal epithelium (i.e. orifice metaplasia) and the lid [22,25,54]. It may be that these combined androgen actions, promoting lipogenesis and suppressing keratinization, are the reason why topical androgens reportedly enhance the synthesis and secretion of meibomian gland lipids, prolong the tear film breakup time, and alleviate dry eye syndrome [44,45].

Testosterone's effects on the female lacrimal and meibomian gland most likely involve an association with saturable, high-affinity and androgen-specific receptors in acinar epithelial cell nuclei. Androgen receptors are members of the steroid/thyroid hormone/retinoic acid family of ligand-activated transcription factors and mediate the classical actions of androgens throughout the body [55,56]. After androgen binding to the receptor, the monomeric, activated hormone-receptor complex associates with an androgen response element in the regulatory region of specific target genes, typically dimerizes with another androgen-bound complex and, in combination with appropriate coactivators and promoter elements, modulates gene transcription [55,56].  In support of this hypothesis, androgen receptors have been shown to exist in lacrimal and meibomian gland epithelial cells [57-63] and androgen activity in these cells may be compromised by androgen receptor mutations or antagonists [22-25,64-69]. Another mechanism of androgen action may involve binding to glandular membrane receptors, stimulation of signal transduction cascades and consequent alteration of gene transcription [56,70]. Testosterone may also act indirectly, by regulating the release of anterior pituitary hormones that may influence the lacrimal and meibomian glands.

Many of the genes modulated by testosterone in female tissues are identical to those regulated by androgens in male lacrimal and meibomian glands. However, some genes are controlled in a sex-specific manner. There were 4 genes downregulated by testosterone in the female, but not male, lacrimal gland. These include lymphocyte antigen 78, which is involved in innate immunity [70], a potassium voltage-gated channel member, and homer protein homolog 3, that helps couple surface receptors to intracellular calcium release [71]. In the meibomian gland, several genes were upregulated in the female, but not male, including prostaglandin (PG) E synthase 2 (catalyzes the conversion of PG H2 to PG E2 [71]), estrogen related receptor α (may modulate the estrogen signaling pathway [71]), and tropomodulin 4 (blocks the elongation and depolymerization of actin filaments [71]). In addition, a series of meibomian gland genes were downregulated by testosterone solely in the female, such as glial fibrillary acidic protein (an intermediate filament [71]), chemokine (C-C motif) ligand 7 (attracts monocytes and eosinophils [71]) and interleukin 1β. Our analyses did not identify any gene that was uniquely and significantly expressed in female, as compared to male, glands.

We were especially interested in comparing the effects of the different sex steroids on gene expression in the lacrimal and meibomian glands. Androgens, estrogens and progestins play essential roles in the health and well-being of both men and women [72,73]. However, these hormones may also have antagonistic effects. An example is the influence of androgens and estrogens on the sebaceous gland. Androgens regulate the development, differentiation and lipid production of sebaceous glands throughout the body [74-78], and many of these actions appear to involve androgen receptors and the control of gene transcription in acinar epithelial cells [78-82]. Conversely, estrogens reduce the size, activity and lipid output of sebaceous glands [74,76,82-86] and for years were used clinically to decrease sebaceous gland function and secretion [74,75,84,85,87,88]. One mechanism proposed for estrogen action is that this hormone induces the release of lysosomal enzymes within sebocytes, resulting in premature cellular destruction and attenuated sebum elaboration [86,89]. Additional mechanisms, though, are that estrogens decrease testosterone uptake, interfere with testosterone's conversion to dihydrotestosterone, and antagonize androgen action in the sebaceous gland [15,83,86]. Indeed, estrogens have been described as the mainstay of treatment to reduce androgen effects on the sebaceous gland [75]. These 'anti-androgen' actions of estrogens are dose-dependent, and may be overridden by exposure to physiological levels of androgens [74,84].

Androgen treatment also causes a significant decrease in the number of estradiol binding sites [89,90], and both hormones antagonize each other's regulation of their own receptor [15], in sebaceous glands. Indeed, some androgen effects are thought to be dependent upon low levels of estrogen [91]. This steroid antagonism is not limited to androgens and estrogens. Androgens and progestins, for instance, may also show opposite effects on the same processes [92].

In the lacrimal and meibomian glands, a number of identical genes were influenced by testosterone, and 17β-estradiol and/or progesterone. The nature of the gene response to these hormones was sometimes similar, but often opposite. One lacrimal gland gene upregulated by 17β-estradiol and downregulated by testosterone is asialoglycoprotein receptor 1, which has been linked to the development of exocrine gland inflammation and dry eye [93-95]. These differential actions could contribute to the estrogen pro-inflammatory [33], and the androgen anti-inflammatory [33-36], effects in lacrimal tissue in Sjögren's syndrome. Genes stimulated by testosterone, but suppressed by 17β-estradiol, in the meibomian gland include secreted acidic cysteine rich glycoprotein (regulates cell growth [71]), vascular endothelial growth factor A (promotes cell migration, among many other actions [71]), matrix metalloproteinase 3 (degrades fibronectin, laminin, gelatins and collagens [71]) and cathepsin K (degrades extracellular matrices [71]). These genes could theoretically be involved in cell maturation, migration and holocrine secretion in the meibomian gland. If so, this would be consistent with a pro-sebaceous effect of androgens and an anti-sebaceous activity of estrogens. Such opposite effects of androgens and estrogens could also involve post-transcriptional [14] and non-genomic [96] pathways.

In summary, our data demonstrate that testosterone, 17β-estradiol and progesterone exert multiple effects on the lacrimal and meibomian glands, and that certain of these sex steroid actions are sex-specific and/or opposite. It is quite possible that these opposing sex steroid effects may play a role in the pathogenesis of dry eye syndrome.
